# Cryo-EM structure of human heptameric pannexin 2 channel

**DOI:** 10.1038/s41467-023-36861-x

**Published:** 2023-03-03

**Authors:** Hang Zhang, Shiyu Wang, Zhenzhen Zhang, Mengzhuo Hou, Chunyu Du, Zhenye Zhao, Horst Vogel, Zhifang Li, Kaige Yan, Xiaokang Zhang, Jianping Lu, Yujie Liang, Shuguang Yuan, Daping Wang, Huawei Zhang

**Affiliations:** 1 Department of Biomedical Engineering, Southern University of Science and Technology, Shenzhen, 518055 China; 2 Shenzhen Institute of Advanced Technology, Chinese Academy of Sciences, Shenzhen, 518055 China; 3 Department of Child and Adolescent Psychiatry, Shenzhen Kangning Hospital, Shenzhen Mental Health Center, Shenzhen, 518020 China; 4 School of Life Sciences, Southern University of Science and Technology, Shenzhen, 518055 China; 5 Interdisciplinary Center for Brain Information, The Brain Cognition and Brain Disease Institute, Shenzhen Institute of Advanced Technology, Chinese Academy of Sciences, Shenzhen, Guangdong 518055 China; 6 Faculty of Life and Health Sciences, Shenzhen Institute of Advanced Technology, Chinese Academy of Sciences, Shenzhen, Guangdong 518055 China; 7 Shenzhen-Hong Kong Institute of Brain Science-Shenzhen Fundamental Research Institutions, Shenzhen, Guangdong 518055 China; 8 Department of Orthopedics, Shenzhen Intelligent Orthopaedics and Biomedical Innovation Platform, Guangdong Provincial Research Center for Artificial Intelligence and Digital Orthopedic Technology, Shenzhen Second People’s Hospital, The First Affiliated Hospital of Shenzhen University, Shenzhen, 518000 China; 9 Guangdong Provincial Key Laboratory of Advanced Biomaterials, Southern University of Science and Technology, Shenzhen, 518055 China

**Keywords:** Structural biology, Electron microscopy, Permeation and transport

## Abstract

Pannexin 2 (Panx2) is a large-pore ATP-permeable channel with critical roles in various physiological processes, such as the inflammatory response, energy production and apoptosis. Its dysfunction is related to numerous pathological conditions including ischemic brain injury, glioma and glioblastoma multiforme. However, the working mechanism of Panx2 remains unclear. Here, we present the cryo-electron microscopy structure of human Panx2 at a resolution of 3.4 Å. Panx2 structure assembles as a heptamer, forming an exceptionally wide channel pore across the transmembrane and intracellular domains, which is compatible with ATP permeation. Comparing Panx2 with Panx1 structures in different states reveals that the Panx2 structure corresponds to an open channel state. A ring of seven arginine residues located at the extracellular entrance forms the narrowest site of the channel, which serves as the critical molecular filter controlling the permeation of substrate molecules. This is further verified by molecular dynamics simulations and ATP release assays. Our studies reveal the architecture of the Panx2 channel and provide insights into the molecular mechanism of its channel gating.

## Abstract (web summary)

Pannexin 2 plays critical roles in many physiological processes but its mechanism remain unclear. Here, authors report the cryo-EM structure of human Panx2 in the open state and identify critical residues for its gating.

## Introduction

Pannexins (Panxs) form large-pore nonselective membrane channels which play an important role in cell communication and homeostasis^1^. These channels mediate the exchange of ions and small molecules such as glutamate, Ca^2+^, ATP and inositol triphosphate 3 (IP_3_) between adjacent cells, as well as between cells and the extracellular matrix^2,3^. There are only three members in the Panx family: Panx1, Panx2 and Panx3. Unlike the relatively well-characterized Panx1 with potential effects on various physiological and pathological functions^4^, few studies have been performed with Panx2 and Panx3^1^. Panx1 is widely expressed in most tissues^5,6^, while Panx2 is predominantly expressed in the central nervous system^6^. In addition, Panx1 and Panx2 have been reported to form heteromeric channels with unknown functions^7,8^, although Panx1 shares only 16% sequence identity with Panx2. Panx2 is the member with the largest sequence of the Panx family and has distinct features. Compared with the 426 residues of human Panx1 and the 392 residues of Panx3, Panx2 consists of 677 residues and has a long distinct C-terminal domain with unknown functions^9^. In addition, Panx1 and Panx3 are readily found at the plasma membrane, while Panx2 is also present on intracellular membranes and fusion with Panx1 C-terminus can increase the plasma membrane localization of Panx2^8,9^. Studies of Panx2 may provide novel insights into the working mechanism of Panx family.

Recent studies have demonstrated the significant roles of the Panx family, especially Panx2, in many diseases including cancer and neurological diseases. Overexpression of Panx2 reduced the growth of glioma cells. This observation linked Panx2 to cancer and indicated that Panx2 might be a tumor suppressor^10^. Additional studies found that Panx2 was downregulated in low-grade glioma, glioblastoma multiforme and other cancer types^11^. Patients with high levels of Panx2 had a longer overall survival time than patients with low levels of Panx2, suggesting that Panx2 may have tumor suppressive properties in the early stage of glioma^10^. Panx2 also participates in physiological processes such as the inflammatory response, energy production, and apoptosis and is also considered to be possibly involved in the pathophysiological processes of neurological and mental diseases^12,13^. For instance, Panx2 is critical for channel activity in cortical neurons and can promote ischemic brain injury^14,15^.

The structure of the Panx1 channel has been determined successfully by different groups in recent years^16–21^. The structure shows the Panx1 channel as a homo-heptamer with the funnel-like cylinder shape. Structural analysis and electrophysiological experiments demonstrated that the C-terminal (amino acids 380–426) of the Panx1 protein was deeply inserted into the center of the channel pore, blocking and closing the channel. The cleavage of the C-terminus during cell apoptosis or by caspase cleavage under experimental conditions leads to the opening of the channel^2,16,22^. However, many cellular events related to the Panx family, such as their activation mechanisms, increased extracellular potassium^23–25^ or cytoplasmic calcium levels^26^, hypoxemia^27^ and membrane stretch and voltage^7,28,29^, remain unknown and require further clarification. Thus, investigations on the structure and function of Panx2 will help comprehensively understand functions of the Panx family.

Here, we present the near-atomic-resolution structure of human Panx2 determined by single-particle cryo-electron microscopy (cryo-EM). The oligomerization state and protomer-protomer interfaces are analyzed. The pore radius and potential constriction sites including N-terminal helix, C-terminal loop and R89 residues are identified. Structural comparison of Panx2 with Panx1 structures in different states reveals that the Panx2 structure adopts an open channel state. Those structural observations are further tested by molecular dynamics (MD) simulations and ATP release assays using different Panx2 constructs. Our results provide insights into the substrate permeability and channel gating mechanism of Panx2 and pave the way for the development of structure-based rational therapies targeting Panx2 for the treatment of various pathological conditions.

## Results

### Structural determination of the human Panx2 channel

**Fig. 1:**
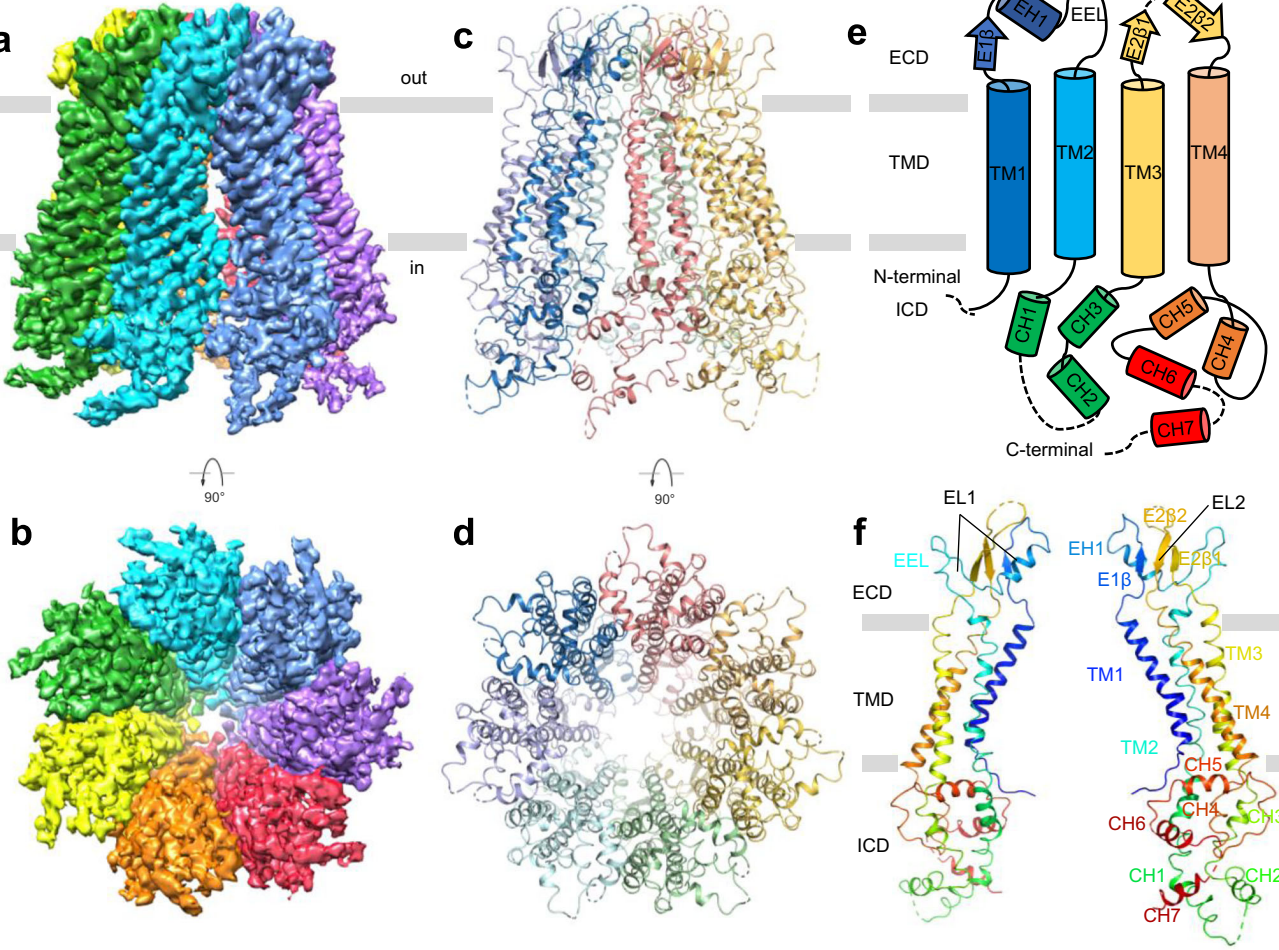
Cryo-EM structure of the human Panx2 channel. Each subunit is colored differently; the membrane boundary is indicated by thick gray lines and out/in represents the extracellular/intracellular space, respectively. Cryo-EM density map of the human N-terminal Panx2 (Panx2-NT) at a resolution of 3.4 Å viewed parallel to the membrane (**a**) and from the intracellular side (**b**). Ribbon representation of the heptameric Panx2 structure viewed parallel to the membrane (**c**) and from the intracellular side (**d**). **e** Schematic representation of the secondary structure of Panx2 protomer. Cylinders and arrows indicate helices and β-strands, respectively. **f** Views of two diagonally opposed protomers colored in rainbow individually, and secondary structural elements are presented. ECD extracellular domain, TMD transmembrane domain, ICD intracellular domain, TMs transmembrane helices, EHs extracellular helices, ELs extracellular loops, EEL extra extracellular loop, CHs C-terminal helices.

We attempted to express the full-length Panx2 protein with different tags fused at its C-terminus in HEK293F cells, but the expression was low. After several trials, we chose the N-terminal domain (Panx2-NT, with amino acids 1-410, Supplementary Fig. 1a) for our studies, to mimic the caspase cleaved Panx2 truncation as reported previously^30^. The Panx2-NT expression level was much higher than that of full-length Panx2 (Supplementary Fig. 1b–d). Purification of Panx2-NT was performed by affinity chromatography and size exclusion chromatography. Panx2 protein was eluted at two peaks (at approximately 10.5 ml and 16.0 ml) on a Superose 6 10/300 column. Panx2 at 10.5 ml peaks was aggregated when viewed under transmission electron microscopy, while Panx2 at 16.0 ml displayed homogeneous mono-dispersity and was quite stable in digitonin-solubilized buffer. Protein purity was further inspected by sodium dodecyl-sulfate polyacrylamide gel electrophoresis (SDS-PAGE), displaying three bands with similar sizes, possibly contributed by slight protein degradation or glycosylation (Supplementary Fig. 1b). Peak fractions were collected and subjected to single-particle cryo-EM analysis. Initial two-dimensional classification indicated a heptameric channel assembly, and three-dimensional reconstruction yielded an electron map at 4.1 Å without imposed symmetry (Supplementary Fig. 2a–c). Because no noticeable differences between subunits from the C1 symmetry map were observed, the final refinements were imposed with C7 symmetry and successfully improved the overall resolution to ~3.4 Å (Fig. 1a–d, Supplementary Fig. 2a, d, and Supplementary Table 1), confirming that Panx2 assembles as a symmetric heptamer. The higher-resolution map with C7 symmetry was used for subsequent model building. The final density map is of excellent quality and shows side-chain densities for most residues (Supplementary Fig. 3a, b), enabling confident model building, which is guided by bulky side chains and assured by disulfide bonds present in the extracellular domain. The refined atomic model fits well into the density with good geometry (Supplementary Figs. 2e, 3a, b, Supplementary Table 1). The amino and carboxyl-terminal sequences (amino acids 1–40 and 389–410) were not resolved in the density map, possibly due to the degradation or dynamic nature of these two portions, and therefore were not used for model building. The final atomic model contained amino acids 41–388, except for several short disordered regions, including an extracellular (amino acids 185-187) and two cytoplasmic segments (amino acids 267–272 and 374–376).

### Structure of the Panx2 protomer

Our Panx2 channel structure is composed of seven identical protomers which are symmetrically assembled around a central axis that form the transmembrane channel pathway. Each protomer can be divided into three domains: an extracellular domain (ECD), a transmembrane domain (TMD), and an intracellular domain (ICD) (Fig. 1e, f). The first extracellular loop (EL1) between helices TM1 and TM2 consists of an extracellular helix (EH1), β-strand E1β and an extra extracellular loop (EEL), and the second extracellular loop (EL2) between helices TM3 and TM4 consists of two β-strands (E2β1 and E2β2). The two extracellular loops EL1 and EL2 formed the compact ECD mainly through two types of interactions. The E1β strand in EL1 forms an intact sheet with the E2β1 and E2β2 strands from EL2, and this interaction is stabilized by two disulfide bonds, one bond between C81 from E1β and C279 from E2β2 and the other bond connecting C99 from helix EH1 and C259 from E2β1 (Supplementary Fig. 4). Helix EH1 forms an angle of approximately 40° with the membrane and is the main component forming the extracellular entrance with the arginine 89 (R89) residue.

The TMD consists of four helices: TM1 and TM2 are pore-lining helices, and TM3 and TM4 are arranged at the periphery of the channel (Fig. 1e, f). The Panx1 channel has an N-terminal helix (NTH) spanning the extracellular half of the TMD^16^. Through protein sequence conservation analysis, AlphaFold structure prediction^31^ and structural alignment with the Panx1 subunit, there could also be an NTH at the N-terminal end of the Panx2 subunit with residues 2-21 (Supplementary Figs. 4, 5e, h), but no clear density for NTH was observed in the cryo-EM map, thus the NTH was not modeled in the final Panx2-NT structure.

The ICD is a helix-rich structure composed of the cytoplasmic loop connecting TM2 and TM3 and the C-terminal residues after TM4 (Fig. 1e, f). Helix CH1 is long and deeply extended into the intracellular space as an extension of TM2, forming the main body of the ICD and is surrounded by six short helices from CH2 to CH7.

### Assembly of the heptameric Panx2 channel

**Fig. 2:**
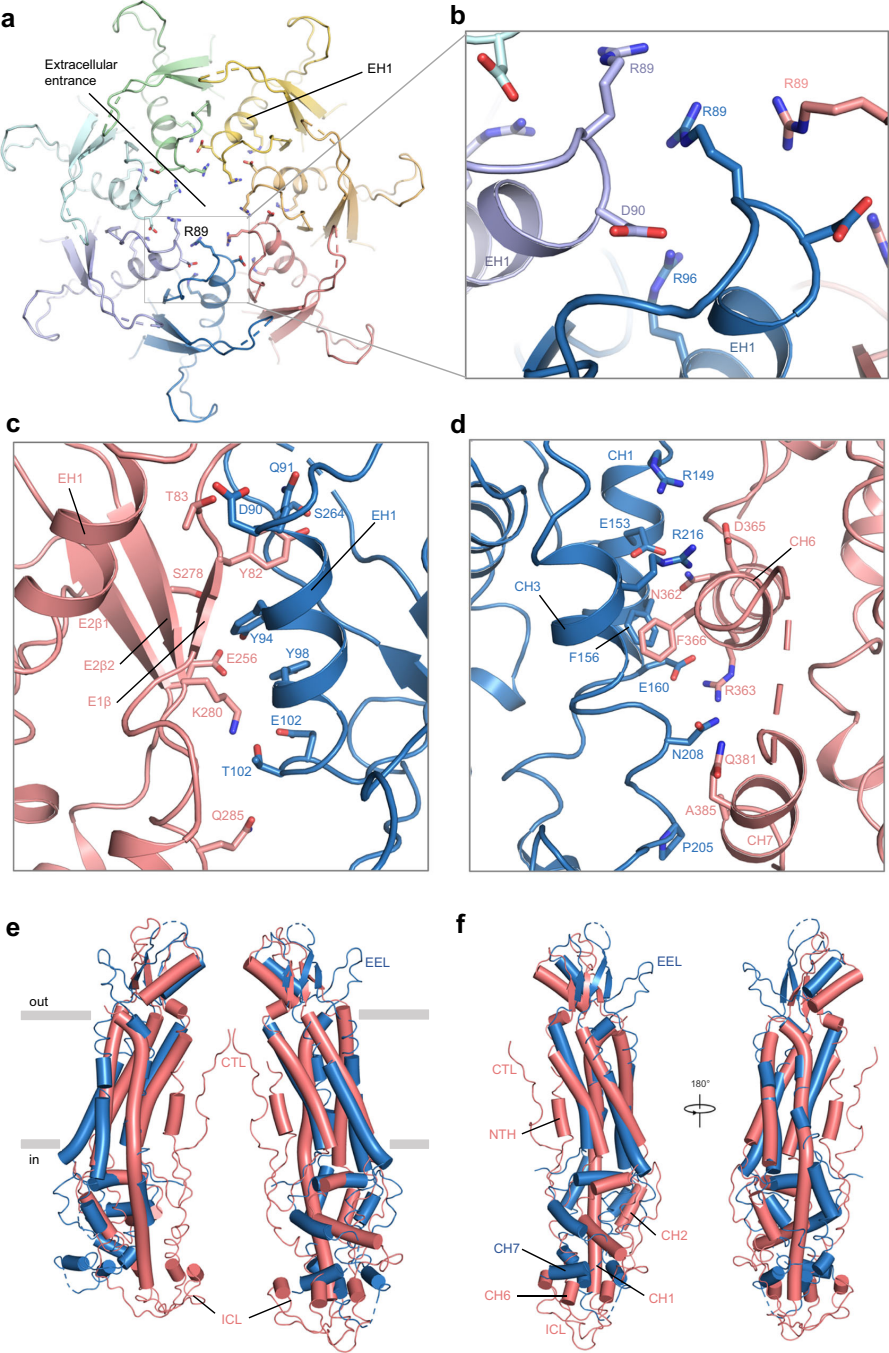
Channel assembly of the Panx2 channel and comparison with the Panx1 channel. **a** Ribbon representation of the extracellular domain (ECD) of the Panx2 structure from each protomer. R89, D90 and R96 of all protomers are shown with side chains. **b** The enlarged view of the extracellular entrance-forming residues of the boxed area in (**a**). The ECD inter-protomer interface (**c**) and the intracellular domain (ICD) inter-protomer interface (**d**). All interacting residues are highlighted and shown in stick representation. **e** Structure comparison between the diagonally opposed protomers of Panx2 and full-length Panx1 (PDB ID: 7DWB). **f** Superposition of the Panx2 protomer and full-length Panx1 protomer. Panx2 was shown in blue, Panx1 was shown in deepsalmon. The N-terminal helix (NTH), intracellular loop (ICL), C-terminal loop (CTL), C-terminal helices CH1, CH2 and CH6 of Panx1 and the extra extracellular loop (EEL) and CH7 of Panx2 are highlighted.

Panx2 is assembled through extensive interactions between the ECD and ICD domains from adjacent protomers, whereas the TMDs only form limited hydrophobic contacts, creating a shallow crevice (Fig. 1a–d). Overall, the Panx2 channel has a contact area of 2023 Å^2^ between adjacent protomers, which is much smaller than that of Panx1 (3920 Å^2^). In the ECD, the most distinctive feature is the seven R89 residues forming a ring in the center of the channel pore at the extracellular entrance (Fig. 2a, b). R89 is conserved in Panx2 from different species such as human, mouse, rat and frog, and may form potential arginine-arginine short-range interactions in a similar manner as previously reported^32,33^ (Supplementary Fig. 4). D90 is sandwiched between R89 and R96 of the adjacent helix EH1, forming possible electrostatic attractions (distance between amino group of R89/R96 and hydroxyl group of D90 is about 5 Å) and further encircling the entrance (Fig. 2b). The side chains of R89 and R96 are more stretching into the pore center, thus, forming the positively charged extracellular entrance. Moreover, multiple contact points from the ECD are widely participating in the protomer-protomer interaction, such as Q91 (EH1)-Y82 (E1β1), E102 (EH1)-Q285 (extension of E2β2) salt bridges and S264 (extension of E2β1)-Y82 (E1β1),Y94 (EH1)-S278 (E2β2) hydrogen bonds (Fig. 2c), which make the ECD region compact and show higher resolution than the other parts (Supplementary Fig. 3a). These specific side-chain interactions between the ECDs further strengthened the pore assembly in the extracellular domain.

On the intracellular side, helices CH1 and CH3 engaged an extensive network of contacts with the C-terminal helices CH6 and CH7 from the adjacent protomer through hydrophobic and hydrophilic interactions (Fig. 2d). The interfaces include residues R149, E153, F156, E160, P205, N208 and R216 from CH1/3, and N362, R363, D365, F366, Q381 and A385 from CH6/7. The resolution of the ICD of the Panx2 channel is relatively lower than that of other parts, indicating the dynamic nature of the ICD (Supplementary Fig. 3a). The structural and functional observations from the Panx1 channel suggest that the cytoplasmic domain plays an important role in regulating channel activity^19,34^. Therefore, the potential regulatory role of ICD in the Panx2 channel requires further investigation.

Since the Panx1 channel structure has been determined by several groups^16–21^, it is necessary and informative to compare the Panx2 structure with that of Panx1. Although the overall structure of Panx2 and Panx1, and their protomers show high similarities (Supplementary Fig. 5a–c), there are some significant differences, especially in the ICD. In the ECD, the Panx2 subunit has an additional extracellular loop (EEL), EL1, of yet unknown function. On the other hand, NTH and CTL are only present in Panx1 but not in Panx2 (Fig. 2e, f, Supplementary Fig. 5d, e). There are additional differences in the ICD: (i), Helices CH3-6 of Panx2 and CH2-5 of Panx1 adopt similar conformations but show an apparent rotation between each other. (ii) Helix CH2 of Panx2 is not present in Panx1, but is replaced by a long intracellular loop (ICL) between helix CH1 and CH2 (CH3 in Panx2) close to the center of the channel pathway. (iii) CH1 and CH7 of Panx2 show different conformations as the corresponding CH1 and CH6 (CH7 in Panx2) of Panx1. Specifically, unlike the straight CH1 of Panx1, CH1 of Panx2 shows significant curvature, and CH6 of Panx1 is rotated almost 90° relative to corresponding CH7 of Panx2, facilitating the deep protrusion of the long C-terminal loop (CTL) after CH6 into the pore funnel of the Panx1 channel. (Fig. 2e, f and Supplementary Fig. 5b–f). The CTL was reported to inhibit Panx1 channel function effectively, and its removal by caspase cleavage, either during apoptosis or in vitro enzymatic cleavage experiments, led to channel activation and ATP release^2,16,22^. The lack of ICL and CTL in the Panx2 subunit indicates that our Panx2 structure may adopt the open conformation (see next section).

### The C-terminal truncated Panx2 adopts an open channel state

**Fig. 3:**
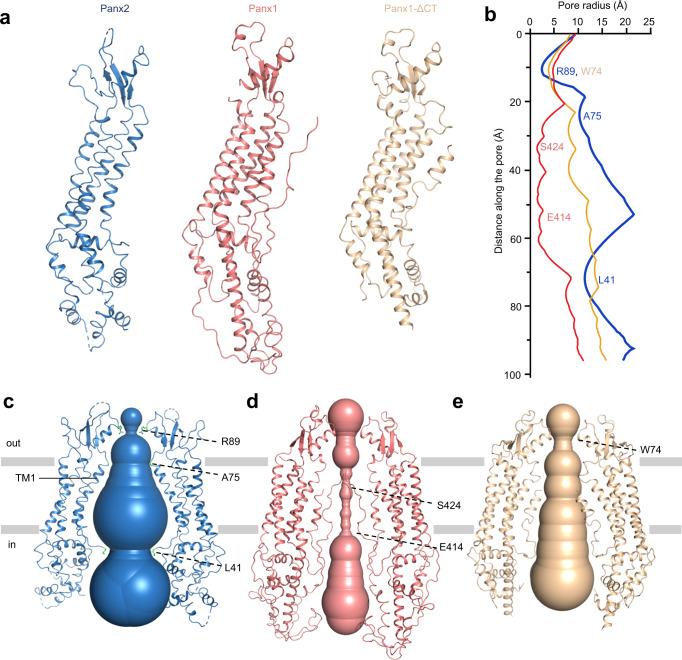
Pore architecture comparison of pannexin channels. **a** Structure comparison of the Panx2 protomer (blue), full-length Panx1 protomer (PDB ID: 7DWB, deepsalmon) and C-terminal truncated Panx1 protomer (Panx1-ΔCT) (PDB ID: 6WBG, wheat). **b** Pore radius of the Panx2, full-length Panx1 and Panx1-ΔCT channel pores calculated with the CAVER program. L41, A75 and R89 of Panx2, E414 and S424 of full-length Panx1 and W74 of Panx1-ΔCT are labeled. Source data are provided as a Source Data file. Channel pore of the Panx2 (**c**), full-length Panx1 (**d**) and Panx1-ΔCT (**e**) structures are shown along two opposing protomers. Selected residues at the constriction sites are shown in stick representation (green) in (**c**). L41, A75 and R89 of Panx2, E414 and S424 of full-length Panx1, W74 of Panx1-ΔCT are indicated by dashed lines.

Pore radius calculations revealed that the permeation pathway was exceptionally wide both in the TMD and ICD and that the construction site was formed by the ECDs (Fig. 3a–c). The narrowest region of the channel pore was formed by a ring of seven R89 residues, each of which was located in the N-terminus of helix EH1, lining the wall of the extracellular entrance with a pore radius of ~3.0 Å (Figs. 2a, b, and 3b, c). For comparison, the corresponding size-selective filter in the Panx1 channel is formed by a ring of seven tryptophan residues W74 (Fig. 3b–e and Supplementary Fig. 4).

Underneath the extracellular entrance, the channel pore in the TMD was predominantly lined by hydrophobic residues from TM1 on the extracellular side (Fig. 3c). The tilt of TM1 in the membrane placed its C-terminal extracellular end (A75) at the narrowest point of the pore in the TMD, with a hydration radius of approximately 10 Å. The N-terminal end of TM1 on the intracellular side is quite far from the central channel pore, but the N-terminal tail (L41) preceding TM1 turned and protruded toward the center of the pore (Fig. 3b, c). Because the 40 N-terminal amino acids were not resolved in the structure of Panx2, the pore dimensions in this region could not be determined. Yet it is reasonable to assume that the NTH is located closely to helices TM1 and TM2 and stays in the pore lumen, since the residue sequences in the NTH between Panx2 and Panx1 are partially conserved, and the NTH of Panx2 predicted by AlphaFold adopts similar conformation as the NTH of Panx1 (Supplementary Figs. 4 and 5e, h).

**Fig. 4:**
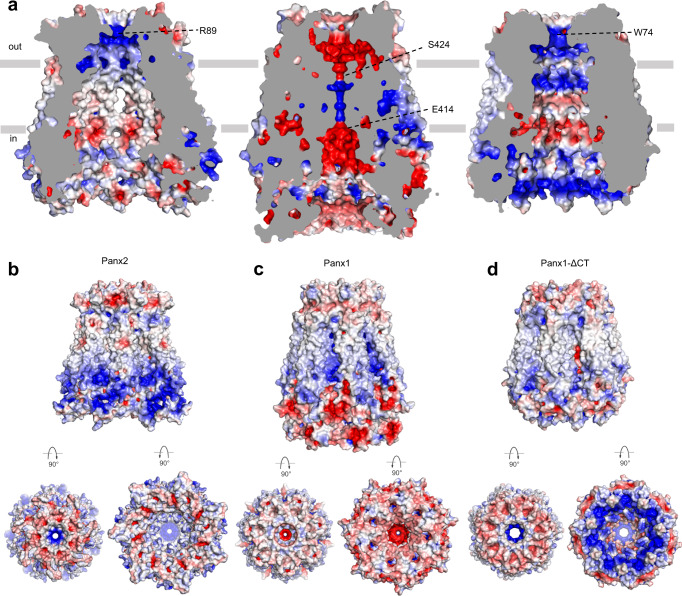
Electrostatic properties comparison of pannexin channels. **a** The interior surface of Panx2, full-length Panx1 and Panx1-ΔCT colored according to the electrostatic surface potential from −5 to +5 kT e^–1^ (red to blue). The cross-section is colored gray. Surface representation of the Panx2 (**b**), full-length Panx1 (**c**) and Panx1-ΔCT (**d**) structures colored by electrostatic surface potential. The side view, top view and bottom view of all structures are presented. R89 of Panx2, E414 and S424 of full-length Panx1, W74 of Panx1-ΔCT are indicated by dashed lines.

**Fig. 5:**
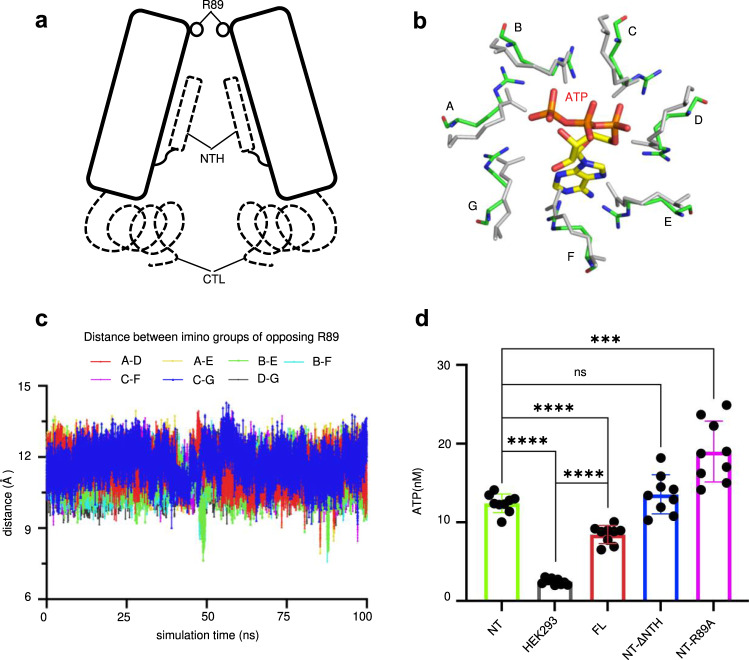
The Panx2 channel adopted an open conformation without the C-terminus and was gated by R89. **a** Schematic diagram of two opposing protomers of Panx2 for demonstrating the three potential portions involved in channel permeation regulation - the residue R89, N-terminal helix (NTH) and C-terminal loop (CTL). The NTH and CTL are drawn with dashed lines. **b** Superposition of the side chains of seven R89 residues of Panx2 before (gray) and after (green) the MD simulations (one frame is captured to display the increased distance between R89 residues). For reference, an ATP molecule is manually placed in the entrance and shown in stick representation. **c** The distances between imino groups of seven paired opposing R89 residues during the MD simulations. Chains are labeled from A to G in (**b**) and (**c**). **d** Normalized results of ATP release assay. The extracellular ATP concentrations measured in adhered HEK293F cells transfected with Panx2-NT (NT), Panx2-FL (FL), Panx2-NT-ΔNTH (NT-ΔNTH), or Panx2-NT-R89A (NT-R89A) plasmid or without plasmid (HEK293). All data were normalized to the NT group except for HEK293 cells. Bars indicate means ± SEMs (*n* = 9 biologically independent dishes of cells). For statistical comparisons to NT or HEK293, the Student’s *t*-test was applied (two-sided). *P* = 0.2334 (NT/NT-ΔNTH). *P* = 0.0002 (NT/NT-R89A). ns, nonsignificant, *P < 0.05, **P < 0.01, ***P < 0.001 and ****P < 0.0001. For (**c**) and (**d**), the source data are provided as a Source Data file.

The Panx1 channel has been proven to have very low permeability for ions and practically no permeability for ATP and small molecules, both by functional and structural studies^16,24,35^. We found that the pore radius of the narrowest position of the Panx2 channel (~3 Å, formed by R89) is larger than that of the Panx1 channel (Panx1-FL) (~1.9 Å, formed by E414 and S424) but also smaller than that of the activated or C-terminal truncated Panx1 channel (Panx1-ΔCT) (~4.4 Å, formed by W74), which permits the chloride/cation and ATP to pass through^16,17^ (Fig. 3d, e). Given the size of an ATP molecule (about 4.4 Å)^16^, it seems difficult to pass through the Panx2 channel. However, considering the strong positively charged environment in the extracellular entrance (Fig. 4a, b), and the potential weak short-range interaction between the adjacent R89 residues^32,33^, a minor conformational change of the long sidechain of arginine might be sufficient for the channel to adopt the ATP (with negative charge) molecule. Moreover, the electrostatic properties inside the channel pore and at the extracellular/intracellular entrance of the Panx1 channel underwent dramatic changes compared with the activated Panx1-ΔCT, which clearly bears more resemblance to the Panx2 channel in terms of the electrostatic potentials (Fig. 4a–d), indicating that the Panx2 channel seems to be in the open conformation. To further test our hypothesis, molecular dynamics (MD) simulations and ATP release assays were performed. The MD simulations indicated that although helix EH1 was quite stable, the side chains of the R89 residues were relatively flexible, and the distance between the imino groups of the two opposing R89 residues constantly fluctuated in the range of 7.5–14 Å, resulting in the pore radius varying from 2.3 to 5.5 Å, which was sufficient for ATP permeation (Fig. 5a–c, Supplementary Fig. 6). Furthermore, ATP release assay confirmed that the Panx2 channel indeed showed permeability for ATP (Fig. 5d). Thus, consistent with our structural observations, the Panx2 channel might be in the open conformation without its C-terminal (Panx2-NT) since the long C-terminal is not included in our structure.

Last, and interestingly, comparing the overall structure of Panx2 with the Panx1 channel, we found that the pore radius of the TMD and ICD of the Panx2 channel is much wider than that of the Panx1 channel or Panx1-ΔCT channel (Fig. 3b–e). Actually, the overall structure of Panx2 is wider and shorter than Panx1, owing to the existence of the EEL and the absence of the ICL and CTL, but this could only explain why the ECD but not the TMD and ICD of the Panx2 channel is relatively wider (Fig. 3e, f, Supplementary Fig. 5a–c). Structural superposition of the protomers from two channels hinted that the ECD of Panx2 was tilted by an additional 14° from the center pore axis compared to the ECD of Panx1 (Supplementary Fig. 5d). However, the ECD of the two channels is stable and of high rigidity with close resemblance revealed by aligning the overall structures, so the actual relative tilting portions might be the TMD and ICD of the Panx2 channel, which could be clearly seen through structural superposition by fixing the ECDs (Supplementary Fig. 5g). This may explain the widening of the channel pore in Panx2, especially in the ICD domain (Fig. 3b–e and Supplementary Fig. 5a–c). Another possibility is that the missing of NTH and CTL region might also contribute to the wider pore size of Panx2 channel.

### Permeation regulation of the Panx2 channel

Our structure implies that the three sites, R89, NTH and CTL, might regulate the ATP/ion permeation of the Panx2 channel (Fig. 5a). The narrowest ring of constriction of the channel formed by R89 may serve as an extracellular selectivity filter controlling the size of permeable molecules (Fig. 3b, c). To test this hypothesis, we further conducted ATP release assays using different versions of Panx2, including Panx2-FL (FL), Panx2-NT (NT), Panx2-NT-ΔNTH (NT-ΔNTH) and Panx2-NT-R89A (NT-R89A) (Supplementary Fig. 1a). The results of these experiments showed that the extracellular ATP concentration of the NT-R89A mutation was significantly increased compared with that of the Panx2-NT, suggesting that the pores formed by the seven R89 residues inhibited the permeation of ATP molecules (Fig. 5d, Supplementary Fig. 7).

For the missing NTH in our Panx2 structure, it is reasonable to assume that it may adopt similar conformation in Panx2 as in Panx1 (Fig. 2f and Supplementary Fig. 5e, h). Taking furthermore into account that the NTH of the Panx2 channel predicted by AlphaFold is longer than that of the Panx1 channel (Supplementary Fig. 5g, h), it is likely that NTH is forming constriction sites in the channel pore in the TMD, especially on the extracellular side of the TMD. However, the difference in ATP concentrations between Panx2-NT and NT-ΔNTH is not significant (Fig. 5d, Supplementary Fig. 7), possibly due to the unexpected conformation adopted by the NTH in Panx2 or the constriction pore formed by the NTH is still wider than that of the R89 residue.

The extremely long and flexible CTL (amino acids 400–677) of the Panx2 channel has no similarities with the short CTL (amino acids 380–426) of the Panx1 channel, and the Panx2 CTL is too long to entirely insert into the channel pore as the Panx1 CTL (Supplementary Fig. 5h), indicating that the CTL of the Panx2 channel may function in a unique manner. In the ATP release assay, the concentrations of ATP were significantly increased in Panx2-NT or Panx2-FL, compared with control group. Further, ATP concentration is also significantly increased in Panx2-NT compared with Panx2-FL. This holds true for either unnormalized or normalized results (with plasma membrane expression levels), suggesting that the Panx2 CTL was partially blocking the permeation of ATP molecules (Fig. 5d, Supplementary Figs. 1c, d, 7). Those data indicate that the CTL may also be involved in the gating of Panx2 channel to transport ATP. In summary, the long CTL may participate in the gating of the Panx2 channel to transport its substrates like ATP molecule.

## Discussion

In this study, we solved the near-atom-resolution cryo-EM structure of the human Panx2-NT structure. The Panx2 structure was assembled as a heptamer adopting a conformation similar to that of the Panx1 structure from the same family. Structural differences are also noted, such as the presence of EEL and the widening pore of TMD and ICD (Figs. 2e, f and 3). In combination with results from cellular experiments, we confirmed that the heptameric Panx2 channel was functional, therefore providing more evidence to demonstrate that Panx2 can form functional channels at the plasma membrane on its own^8^. In our structure, except for three short loops between β-sheet E2β1 and E2β2 (amino acids 185-187), helix CH1 and CH2 (amino acids 267-272), helix CH6 and CH7 (amino acids 374-376) and the missing NTH (amino acids 1–40), the structure of the N-terminal Panx2 channel was almost completely modeled (Fig. 2e, f). The considerably long CTL of the Panx2 channel is not included in our structure. Although the CTL is predicted to be intrinsically disordered, we could still not rule out the possibility of forming an ordered region at least partially, similar to the CTL of the Panx1 channel^16^.

Compared to the Panx1 channels in both inactivated and activated states, we found that the pore radius distribution and the electrostatic properties of the Panx2 channel were comparable to those of the activated Panx1-ΔCT channel (Figs. 3 and 4), indicating that the Panx2 channel adopts an open conformation, which was further strengthened by the MD simulations and ATP release assays (Fig. 5 and Supplementary Fig. 7). Furthermore, the pore radius calculation revealed several constriction sites in the channel pore including R89, NTH and CTL. Taken together with the results from cellular experiments, the R89 residue was identified as the most critical selectivity filter controlling the permeation of molecules (Figs. 2 and 5). Although the NTH is not modeled in our structure which may affect the pore radius distribution, it is still possible that it does not form the narrowest site in the channel pore, as our cellular experiments showed that mutation of R89 could affect the permeability of Panx2 more significantly than that of NT-ΔNTH (Figs. 2e, f, and 5d). This indicates that constriction pores formed by the NTH might possess an appropriately wider pathway than that of R89. Finally, Panx2 was shown to be a substrate for caspases in vitro, and predicted to harbor the caspase cleavage site in the CTL^30^. Consistent with increased extracellular ATP concentration after the removal of the CTL, Panx2 may play a similar function to that of Panx1, releasing molecules such as ATP during cell apoptosis.

Another unanswered vital question is whether the Panx family members have the ability to form gap-junction channels. Unlike other similar four-transmembrane-domain arranged large-pore channels, including connexins, volume-regulated anion channels (VRACs), calcium homeostasis modulators (CALHMs) and Panxs’ invertebrate homolog innexins^36–44^, Panxs were generally thought to be unable to form gap-junction channels due to their N-glycosylation^45–47^, and a glycosylation-deficient mutant (N255A) of Panx1 has been identified to form the gap-junction-like structure^16^. Nevertheless, it is noteworthy that the gap-junction-like structure may not represent a normal physiological conformation of the channel^48^. Consistent with our structure, Panx2 was also found to be N-glycosylated on N86 (extra density observed near N86, Supplementary Figs. 3b, 4a) and assembled as a single heptamer similar to glycosylated Panx1 structures^16–21^. In addition, two-dimensional classification of Panx2 particles did not show any gap-junction-like features (Supplementary Fig. 2c).

The opening of nonselective large-pore channels in the neuronal membrane has been reported to be the cause of several cellular events, such as the loss of essential metabolites, including glutathione and ATP, anoxic depolarization and the release of toxic compounds, all of which promote neuronal cell death^15^. The *Panx1*- and *Panx2*-knockout mice showed protective effects against ischemic brain damage, indicating that Panx1 and Panx2 channels may show permeability properties for the aforementioned molecules, which is consistent with the increased extracellular ATP concentration of Panx2-FL compared with the control (Supplementary Fig. 7). The increased extracellular potassium concentration in cerebral ischemia^23^ and abundant reactive oxygen species and NO in ischemic brain tissue^49^ may also be related to the opening of the Panx2 channel. Capturing different functional states of the Panx2 channel, especially of its NTH and CTL domains, will further help the elucidation of its unique roles in those cellular events.

## Methods

### Cloning, cell culture and transfection

Full-length and truncated DNA fragments (Panx2-NT, Panx2-ΔNTH) encoding *H. sapiens* PANX2 (NCBI:NP_443071.2) were amplified from cDNA and subcloned into the mammalian expression vector pSecTag2B with a C-terminal FLAG tag. Mutations were introduced by site-directed mutagenesis and confirmed with Sanger sequencing. HEK293F cells were cultured in SMM 293T-II medium (Sino Biological Inc., M293TII-1) under 8% CO_2_ in an incubation shaker (Shanghai Zhichu) at 37 °C. Target protein was expressed heterogeneously by transient transfection. Briefly, for 1 liter culture of HEK293F cells, 1 mg plasmid and 4 mg 25-kDa linear polyethylenimines (PEIs) (Polysciences, 23966-1) were preincubated for 10 min in 25 ml fresh medium, then the two media were mixed for further incubation for 20 min prior to adding the mixture to cells. The transfected cells were cultured for 72 h before harvest by centrifugation. Panx2-FL and all mutants (Panx2-NT, Panx2-NT-ΔNTH, and Panx2-NT-R89A) were transfected using the same method. The information on primers of all clones is listed below:

Panx2-FL, F: 5′-ACTCCAGTGTGGTGGAATTCTATGCACCACCTCCTGGAGCAG-3′;

Panx2-FL, R: 5′-TGTTCGGGCCCTCCTCGAGCAAACTCCACAGTACTCAC-3′;

Panx2-NT, F: 5′-CGCCAACGCTCGAGGAGGGCCCGAACA-3′;

Panx2-NT, R: 5′-CTCGAGCGTTGGCGCTGGGGTCCACGGT-3′;

Panx2-NT-ΔNTH, F: 5′-TTCTATGCTTCTGCAGCTGAAGCTGGAG-3′;

Panx2-NT-ΔNTH, R: 5′-GCAGAAGCATAGAATTCCACCACACTGG-3′;

Panx2-NT-R89A, F: 5′-CACGGCCGACCAGGCGCTGTACGCCCGC-3′;

Panx2-NT-R89A, R: 5′-CTGGTCGGCCGTGAAGTTGTGCGGGGTGTA-3′.

### Protein preparation

For each batch of protein purification, 8 liters of transfected HEK293F cells were harvested by centrifugation at 3000 × *g*. Cell pellets were resuspended in lysis buffer containing 50 mM Tris, pH 7.5, 150 mM NaCl, 0.2 mM benzamide, and 0.3 mM PMSF and disrupted by high-pressure milling (JNBIO Inc.). Cell membranes were solubilized in lysis buffer containing 1% (wt/vol) N-dodecyl-β-D-maltoside (DDM, Anatrace, D310S) for 12 h with stirring at 4 °C. Solubilized Panx2 protein was separated from the insoluble fraction by centrifugation for 1 h at 30,000 × *g* and incubated with 2 ml of anti-FLAG M2 agarose beads (Sigma-Aldrich, M8823) for 2 h at 4 °C. The resin was then washed with 10 column volumes of buffer containing 50 mM Tris pH 7.5, 150 mM NaCl, 0.2 mM benzamide, 0.3 mM PMSF and 0.2% DDM. Resin was then incubated with 4 column volumes of buffer containing 50 mM Tris pH 7.5, 150 mM NaCl, 0.2 mM benzamide, 0.3 mM PMSF, 0.2% DDM and 0.5 mg/ml 3× FLAG peptides (GenScript, C967SHE300-3) for 1 h with stirring at 4 °C. The flow-through was then collected and concentrated using a 100 kDa concentrator (Millipore) and further purified on a Superose 6 gel filtration column (Cytiva) in 50 mM Tris pH 7.5, 150 mM NaCl and 0.02% glyco-diosgenin (GDN, Anatrace, GDN101). The fractions corresponding to the heptameric channels were collected and concentrated to ~10 mg/ml for cryo-EM grid preparation. The final protein concentration was measured by UV absorbance at 280 nm.

### Cryo-EM sample preparation and data collection

The cryo-EM grids were prepared using Vitrobot Mark IV (Thermo Fisher) operated at 4 °C and 100% humidity. Quantifoil holey carbon 300 mesh 1.2/1.3 Cu grids were glow discharged for 50 s. Then, 4 μl sample aliquots at a concentration of approximately 10 mg/ml were applied onto the glow-discharged grids. After waiting for 3 s, the grids were blotted for 2.5 s and plunged into precooled liquid ethane for flash freezing. The cryo-EM grids were screened, and the qualified grids were used for data acquisition using a Titan Krios microscope (Thermo Fisher) operated at 300 kV and equipped with a Gatan K3 Summit detector and GIF Quantum energy filter. Images were automatically collected using SerialEM with a slit width of 20 eV on the energy filter and in the super-resolution counting mode at a nominal magnification of ×130,000 with a calibrated pixel size of 0.92 Å and a nominal defocus value ranging from −1.0 to −2.5 μm. Each movie stack was exposed for 1.45 s with an exposure time of 0.045 s per frame, resulting in a total of 32 frames per stack, and the total dose for each stack was approximately 50 e/Å^2^.

### Image processing and map calculation

Recorded movies were first motion-corrected with MotionCor2^50^ and then subjected to contrast transfer function (CTF) estimation using CTFFIND-4.1^51^. Low-quality images were manually removed from the datasets. For the Panx2 dataset, 1,266 particles were manually picked in Relion-3.1.2^52^ and processed by 2D classification using Relion-3.1.2. Two rounds of 2D classifications were performed to exclude noise and other bad particles. Selected particles were used to generate an initial three-dimensional model as a template for automated particle picking by Gautomatch-v0.56 (http://www.mrc-lmb.cam.ac.uk/kzhang/). Template-based automatic picking resulted in 2,297,566 particles from 7,940 selected micrographs. Particles were extracted using a box size of 288 pixels and imported into cryoSPARC-3.3.1^53^. Then, particles were subjected to two-dimensional classification with a mask diameter of 260 Å. Low-quality particles were discarded by performing two rounds of two-dimensional classification. The resulting good two-dimensional classes containing 396,420 particles were selected to generate an ab initio reconstruction for heterogeneous refinement in cryoSPARC-3.3.1. After heterogeneous refinement, one class showing intact channel features (108,265 particles) was selected and subjected to nonuniform refinement with C1 symmetry, yielding an overall resolution of 4.0 Å. The final round of nonuniform refinement was performed with C7 symmetry applied to further improve the resolution to 3.4 Å. Local resolution estimates were calculated in cryoSPARC-3.3.1^53^.

### Model building and coordinate refinement

A predicted model of human Panx2 was downloaded from the AlphaFold Protein Structure Database (https://alphafold.ebi.ac.uk/entry/Q96RD6). The model was fitted into the cryo-EM density map using map_to_model in Phenix^54^. The TM1-4, EH1 and CH1-5 helices as well as the β-sheet containing E1β1, E2β1 and E2β2 were further adjusted to fit the density, and the remaining model was manually built in COOT^55^. Bulky side chains and disulfide bonds were utilized to facilitate sequence registration. Cycles of model building in COOT and refinement using real_space_refine in PHENIX^54^ were performed to acquire the final refined model. The final model consists of amino acids from positions 41–184, 188–266, 273–373, and 377–388, and the rest of the residues are missing. The final refined models were validated using MolProbity^56^. The solvent accessible contact interface is measured by InterProSulf^57^. The protein contact interface was measured using the InterfaceResidues script in PyMOL (https://pymol.org/2/). Electrostatic potential was calculated using the APBS plugin embedded in PyMOL. Pore dimensions were calculated using the program CAVER^58^. Structural figures were prepared using USCF Chimera^59^ and PyMol.

#### ATP release assay

Extracellular ATP was measured by the luciferin-luciferase reaction. Briefly, HEK293F cells (Thermo Fisher) were grown in Erlenmeyer flasks (BioFit) and transiently transfected at a density of ~2 × 10^6^ cells per ml with Panx2 vectors using polyethylenimines (PEIs) (Polysciences, 23966-1). Cells without transfection of Panx2 vectors were used as controls. After 24 h, transfected cells were transferred to 6-well plates (Corning Costar) and allowed to adhere adequately for 16 h. Well-adhered cells were washed with NaCl-KCl buffer (140 mM NaCl, 3 mM KCl, 1.5 mM Na_2_HPO_4_, 1 mM MgSO_4_, 2 mM CaCl_2_, 10 mM glucose and 10 mM HEPES-NaOH, pH 7.5) twice and incubated in NaCl buffer (143 mM NaCl, 1.5 mM Na_2_HPO4, 1 mM MgSO_4_, 2 mM CaCl_2_, 10 mM glucose and 10 mM HEPES-NaOH, pH 7.5) for 1 h. Supernatants were then transferred to a 96-well black microplate (Corning Costar) and mixed with an Enhanced ATP Assay Kit (Beyotime, S0027) with a prediluted working solution at a ratio of 1:1 (v:v). Data were recorded on a microplate reader (BioTek) with software Gen5. The ATP concentration was calculated from the reading luminance based on the pre-generated ATP standard curve. All ATP concentrations of full-length Panx2 and truncated or mutated Panx2 were normalized to that of Panx2-NT. Signals were normalized with the plasma membrane expression of Panx2 as described below.

#### Normalization of plasma membrane expression of Panx2 using Western Blot

HEK293T cells (10 cm dish) were transfected with different Panx2 plasmids and cultured for 3 days. Cells were washed gently by B buffer (PBS + 0.5 mM CaCl_2_ + 0.5 mM MgCl_2_) at room temperature (RT), then gently incubated with B buffer supplemented with 0.5 mg/ml succinimidyl-6-(biotinamido)hexanoate (NHS-LC-Biotin, Thermo Fisher Scientific, 21336) at RT for 20 min. After that, cells were quickly washed 2 times by 100 mM glycine in B buffer and further washed 2 times by B buffer. Then cells were resuspended and lysed in 0.5 ml RIPA buffer at 4 °C. After incubation for 1 h with stirring at 4 °C, supernatant was separated from the insoluble fraction by centrifugation for 20 min at 14,000 × *g* and 50 µl was kept as “Whole cell”. The rest supernatant was mixed with RIPA-washed streptavidin resins (50 µl) and incubated for 1 h at 4 °C. Then streptavidin resins were collected and washed by RIPA buffer for 3 times. At last, the proteins were eluted by Sodium Dodecyl-Sulfate (SDS) buffer with 50 µl and kept as “Plasma membrane”.

The samples of different Panx2 proteins were applied and separated on 12% Tris-glycine SDS-PAGE gels. The protein was transferred to Immuno-Blot PVDF (Bio-Rad) and then blocked in TBST containing 5% bovine serum albumin (BSA, Sigma-Aldrich, A2153) for 1 h at room temperature. The membranes were incubated at 4 °C overnight with primary anti-FLAG antibodies (Sino Biological Inc., Monoclonal Mouse IgG2a Clone #13, 109143-MM13, 1:1000) diluted in TBST with 5% BSA, followed by horseradish peroxidase (HRP)-linked secondary antibodies (Santa Cruz Biotechnology, sc-516102, 1:5000) for 2 h at room temperature. The membranes were then washed with Amersham ECL Prime Western blotting detection reagent (Cytiva,45-002-401) and exposed using a ChemiDoc MP imaging system (Bio-Rad). The western blot results were quantified using ImageJ. The plasma membrane expression level of Panx2 was normalized using the intensity ratio of “Plasma membrane” and “Whole cell”. The signals from ATP release assay were normalized according to the plasma membrane expression of different Panx2 versions. The FLAG tag antibody was validated by the manufacturer against the DYKDDDDK-tag using western blot, enzyme-linked immunosorbent assay, flow cytometry and immunocytochemistry/immunofluorescence and in multiple expression systems (including *Escherichia coli*, HEK293, Chinese hamster ovary cells and High Five cells).

### Molecular dynamics simulations

Schrodinger software was used to prepare the Panx2 model, including building the missing loop (amino acids 185–187, 267–272, and 374–376) via the Prime ^60^, adding protons to all amino acids via PROPKA^61^, and energy-minimizing the system with the OPLS4 force field^62^ to ensure that no positional conflicts were in the model. Subsequently, a web-based platform for generating the inputs for molecular dynamics named CHARMM-GUI^63^ was applied to build the system. The N- and C-termini of the model were treated as amino termini with a positive charge and carboxyl termini with a negative charge, respectively, as they were freely exposed to the solvation. Two intramolecular disulfide bonds (C81–C279, C99–C259) identified in the experimental structures were created for each chain. The state of the protein after the above processes was regarded as its initial geometry in our simulations. The orientation of the protein and the position of lipid bilayers were determined by the Positioning of Proteins in Membranes (PPM) 2.0 server^64^ and checked manually. The lipid bilayers with the 1-palmitoyl-2-oleoyl-sn-glycero-3-phosphocholine (POPC) model and water molecules with the TIP3P model were generated, resulting in a 120 × 120 × 165 Å box with 200 POPC molecules. The pore water molecules (water in the ion channel) were also added based on the channel geometry. Finally, the entire system was neutralized with 150 mM NaCl.

GPU-accelerated Gromacs 2021^65^ was used to perform the molecular dynamics simulation and CHARMM36m force field^66^ for the molecules. The MD simulation consisted of energy minimization, pre-equilibration and production simulations. The system was first energy minimized with the steepest descent algorithm while keeping 4000 kJ/(mol nm^2^) force constant on backbone atoms and ligand atoms, 2000 kJ/(mol nm^2^) force constant on side-chain atoms and 1000 kJ/(mol nm^2^) force restraint on lipid atoms. Then, six-step pre-equilibration simulations (0.6 ns, 0.6 ns ps, 1 ns, 1 ns, 1 ns, and 1 ns) were carried out, where restraint was reduced slowly (4000, 2000, 1000, 500, 300, 0 kJ/(mol nm^2^) on the backbone and ligand atoms, 2000, 1000, 500, 200, 50, 0 kJ/(mol nm^2^) on side-chain atoms, and 1000, 400, 400, 200, 40, 0 kJ/(mol nm^2^) on lipid atoms) to relax the system. Finally, a production simulation was performed for 100 ns using Langevin thermostat, with a constant temperature of 310 K and a constant pressure of 1 atm. Periodic boundary conditions (PBCs) were introduced during the all molecular dynamics simulations. In all steps, the time step was 2 fs, and atomic coordinates were written every 5 ps. After the MD simulation, root mean squared deviations, root mean squared fluctuations and distances between atoms were analyzed by Gromacs. Details of MD simulations have also been deposited in github at [https://github.com/shiyu-wangbyte/panx2-simulation].

#### Statistical analysis

Each data point of the ATP release assay was measured in five groups, and each group was repeated three times. The minimum and maximum of every measurement were removed. Error bars represent SEM. Regression and statistical analyses were carried out using the program GraphPad Prism 9 (GraphPad Software Inc.). Differences in the mean values of paired data points were evaluated with Student’s *t* test (two-sided).

#### Cell line

HEK293F cells (Thermo Fisher, R79007) and HEK293T cells (Thermo Fisher, K1711) were used in this study. The cell line was not authenticated or tested for mycoplasma contamination.

### Reporting summary

Further information on research design is available in the Nature Portfolio Reporting Summary linked to this article.

## Supplementary information

Supplementary Information

Peer Review File

Reporting Summary

## Data Availability

The data that support this study are available from the corresponding authors upon request. The cryo-EM map of human Panx2 was deposited in the Electron Microscopy Data Bank with accession code EMD-33276 (Cryo-EM structure of human pannexin 2). Atomic coordinates for the human Panx2 structure have been deposited in the Protein Data Bank with accession code 7XLB (Cryo-EM structure of human pannexin 2). The source data underlying Figs. 3b, 5c, d and Supplementary Figs. 1b, d, 6a, b, 7 are provided as a Source Data file. Atomic coordinates for the human Panx1 structures could be accessed with PDB codes 6WBG and 7DWB. A computed model of human Panx2 was downloaded from the AlphaFold Protein Structure Database with ID: AF-Q96RD6-F1. Details for molecular dynamics simulations has been deposited in github at [https://github.com/shiyu-wangbyte/panx2-simulation]. Source data are provided with this paper.

## Source data

Source Data
