# Supplementary material for: Cryo-EM structure of human heptameric pannexin 2 channel
Source: Nat Commun. 2023 Mar 3;14:1118. doi: 10.1038/s41467-023-36861-x (PMC9984531; doi:10.1038/s41467-023-36861-x)
Supplement: Supplementary file 1 — Supplementary Information [file 41467_2023_36861_MOESM1_ESM.pdf]

Supplementary Information For

Cryo-EM structure of human heptameric pannexin 2 channel

Hang Zhang[1], Shiyu Wang[2], Zhenzhen Zhang[1], Mengzhuo Hou[1], Chunyu Du[3], Zhenye Zhao[2], Horst Vogel[2], Zhifang Li[1], Kaige Yan[4], Xiaokang Zhang[5,6,7], Jianping Lu[3], Yujie Liang[3], Shuguang Yuan[2,*], Daping Wang[1,8,*], Huawei Zhang[1,9,*]

[1]Department of Biomedical Engineering, Southern University of Science and Technology, Shenzhen, 518055, China.

[2]Shenzhen Institute of Advanced Technology, Chinese Academy of Sciences, Shenzhen, 518055, China.

[3]Department of Child and Adolescent Psychiatry, Shenzhen Kangning Hospital, Shenzhen Mental Health Center, Shenzhen 518020, China.

[4]School of Life Sciences, Southern University of Science and Technology, Shenzhen, 518055, China.

[5]Interdisciplinary Center for Brain Information, The Brain Cognition and Brain Disease Institute，Shenzhen Institute of Advanced Technology, Chinese Academy of Sciences, Shenzhen, Guangdong 518055, China.

[6]Faculty of Life and Health Sciences, Shenzhen Institute of Advanced Technology, Chinese Academy of Sciences, Shenzhen, Guangdong 518055, China.

[7]Shenzhen-Hong Kong Institute of Brain Science-Shenzhen Fundamental Research Institutions, Shenzhen, Guangdong 518055, China.

[8]Department of Orthopedics, Shenzhen Intelligent Orthopaedics and Biomedical Innovation Platform, Guangdong Provincial Research Center for Artificial Intelligence and Digital Orthopedic Technology, Shenzhen Second People's Hospital, The First Affiliated Hospital of Shenzhen University, Shenzhen,518000, China.

[9]Guangdong Provincial Key Laboratory of Advanced Biomaterials, Southern University of Science and Technology, Shenzhen, 518055, China.

[*]Correspondence: Shuguang Yuan: shuguang.yuan@siat.ac.cn or Daping Wang: wangdp@mail.sustech.edu.cn or Huawei Zhang: zhanghw@sustech.edu.cn

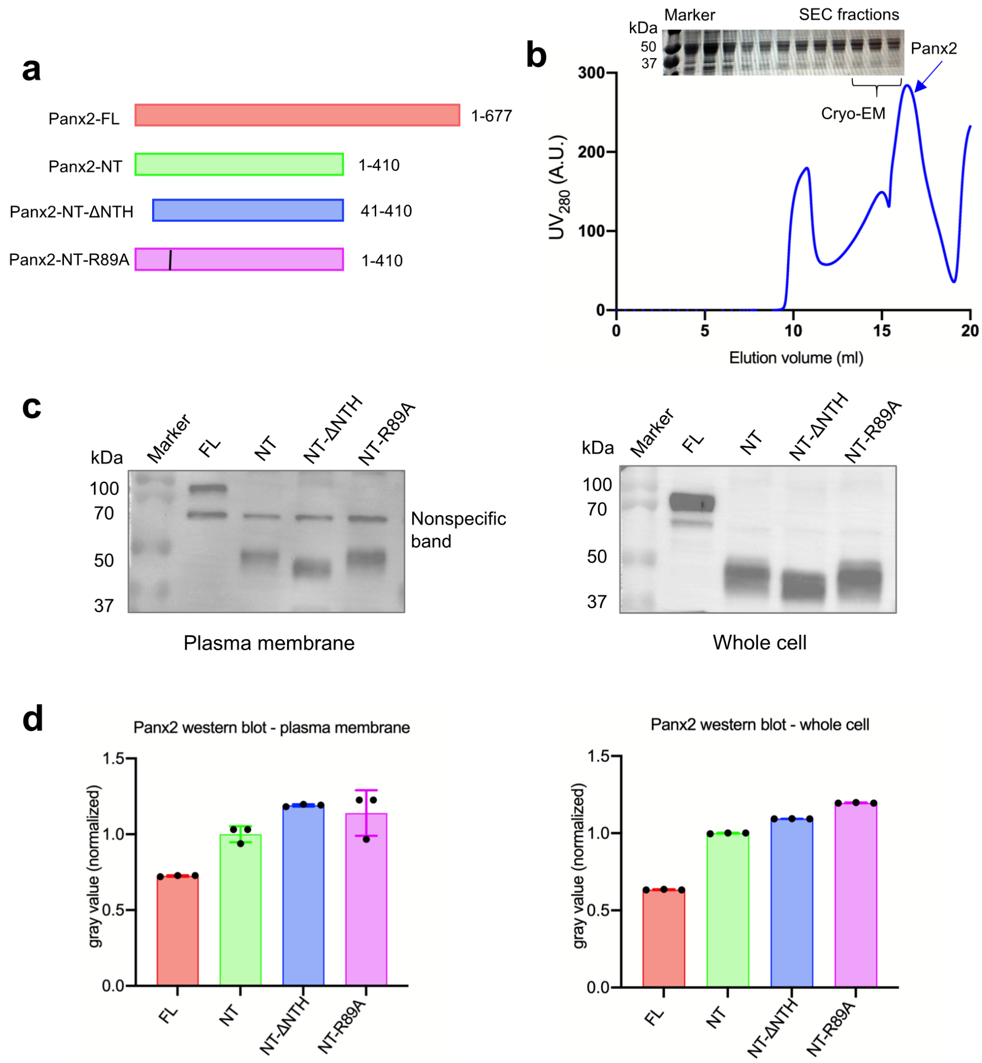

Fig. S1 Protein purification, expression analysis of different versions of Panx2.

a, Schematic diagram of full-length Panx2 and its different truncations or mutations. **b,** Size-exclusion chromatography (SEC) profile of Panx2-NT purification and sodium dodecyl-sulfate polyacrylamide gel electrophoresis (SDS-PAGE) analysis of eluted fractions. The purification was repeated for 4 times with similar results. A.U.: arbitrary units. **c, d,** Representative western blot (**c**) and statistics analysis to show the expression level of the plasma membrane or whole cell of full-length Panx2 and its different truncations or mutations (**d**). Bars indicate means ± SEM (n=3 biologically independent samples). Source data are provided as a Source Data file for panel **b** and **d**. kDa: kilodaltons. FL: full-length Panx2. NT: N-terminal domain of panx2. NTH: N-terminal helix.

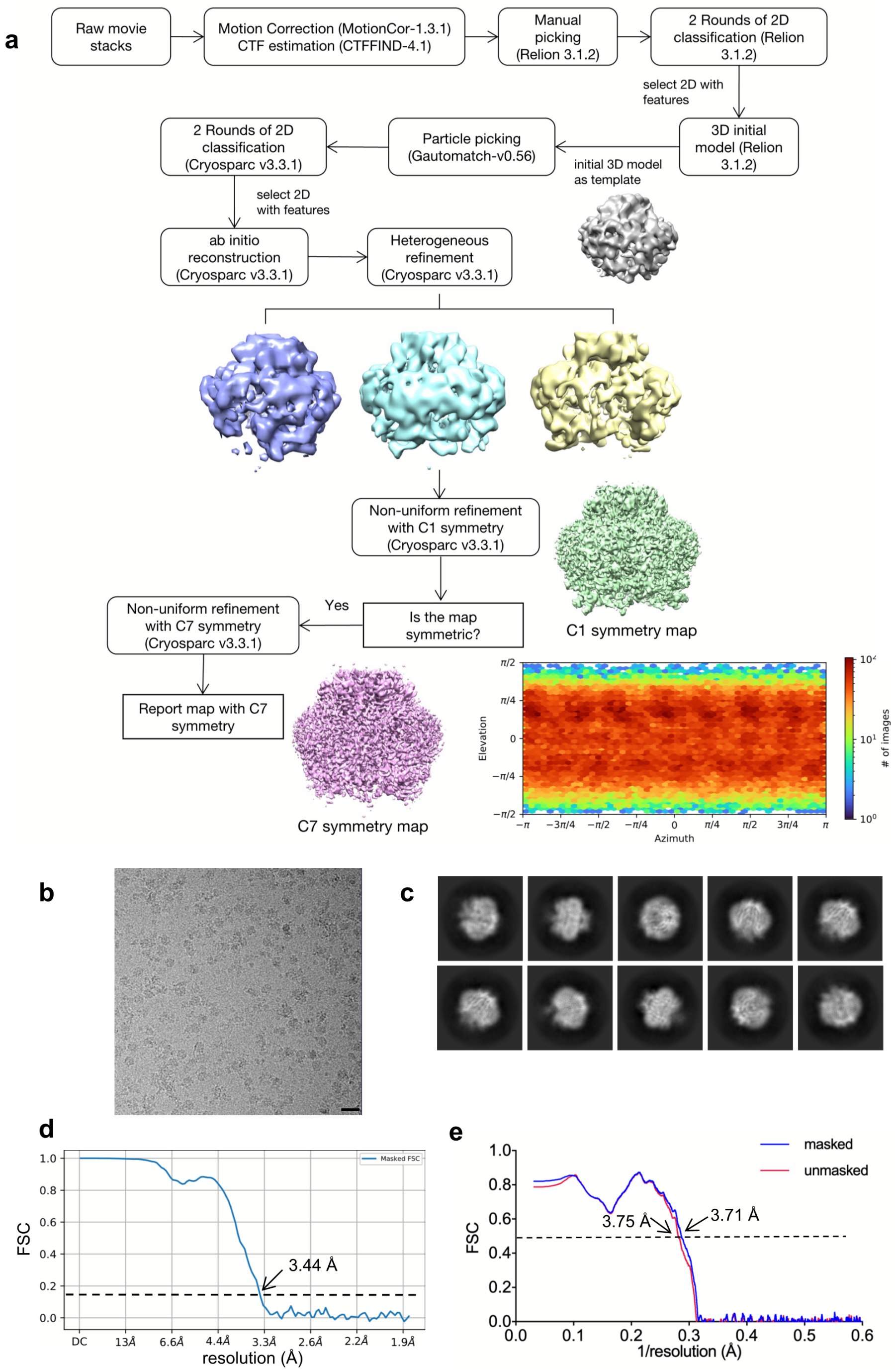

Fig. S2 Workflow of cryo-EM data processing, representative micrographs, 2D class averages and Fourier shell correlation curve.

a, Data processing workflow of Panx2 cryo-EM analysis. A detailed description of the data analysis procedure can be found in Methods section. The angular distribution of particles used in the final reconstruction is shown alongside the final map. **b**, Selected micrograph from 8,039 micrographs is presented with pixel size of 0.92 Å. scale bar, 20 nm. **c,** Selected two-dimensional class averages are shown with a box size of 320 pixels. **d,** Gold-standard Fourier shell correlation (FSC) of the final map in cryoSPARC-3.3.1, and the map resolution is determined by the gold-standard 0.143 criterion. **e,** Fourier shell correlation between the refined model and the masked or unmasked map at 0.5 cut-off.

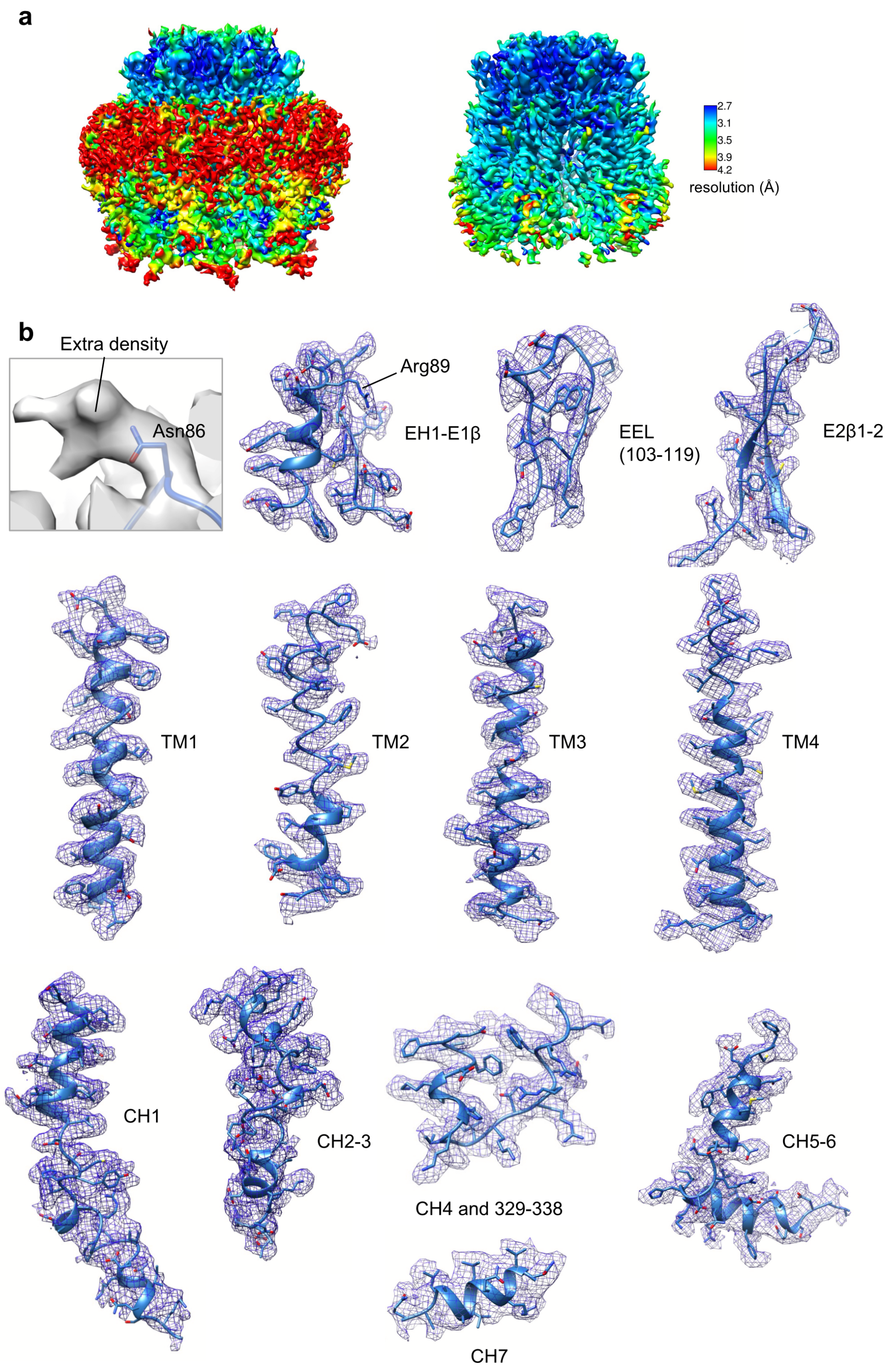

Fig. S3 Local resolution estimation and representative densities.

a, Cryo-EM density map with different levels is colored by local resolution. Colors correspond to resolutions: <2.7 Å (bule); 2.7-3.1 Å (bule-cyan); 3.1-3.5 Å (cyan-green); 3.5-3.9 Å (green-yellow); 3.9-4.2 Å (yellow-red); >4.2 Å (red). **b,** Representative densities are shown for secondary structure elements and loops of Panx2 structure. Arg89 is indicated. Extra density near Asn86 is shown.

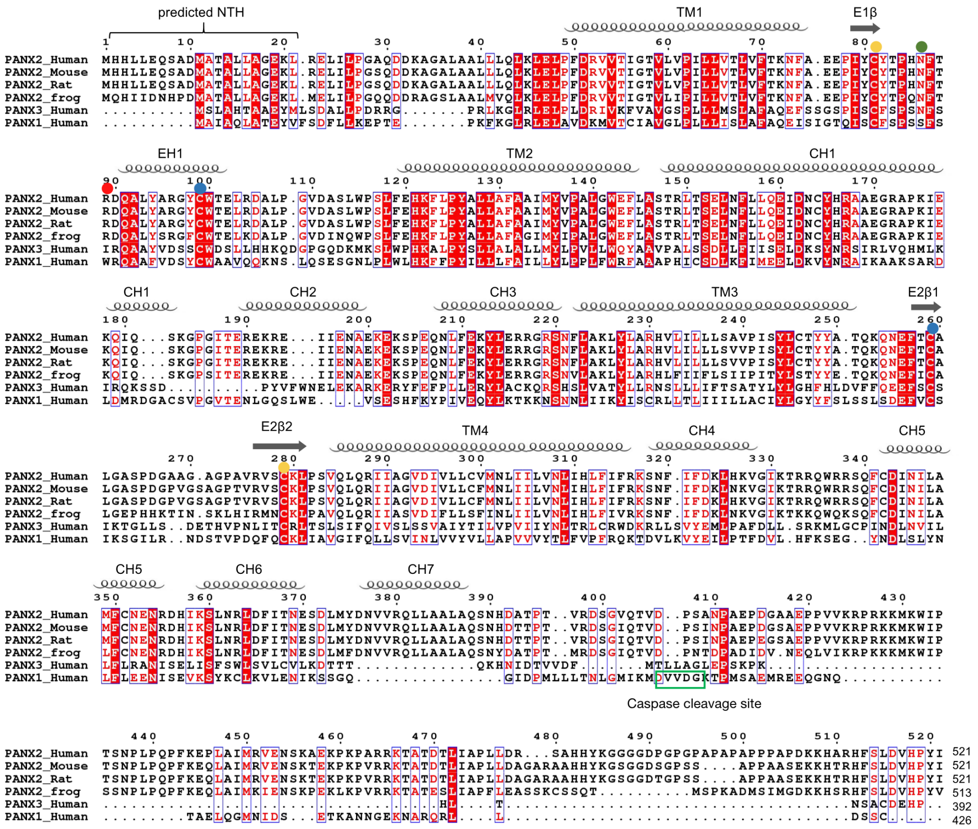

Fig. S4 Secondary structure arrangement and sequence alignment.

The protein sequence alignment of Panxs family. Secondary structures based on the Panx2 structure model are labeled. The cysteine residues forming the extracellular disulfide bonds are highlighted by two paired yellow and blue dots, respectively. The R89 forming the extracellular entrance is marked with a red dot. The N86 glycosylation site is marked with a green dot. The partial C-terminal residues (amino acids 522-677) of Panx2 sequences are omitted for clarity.

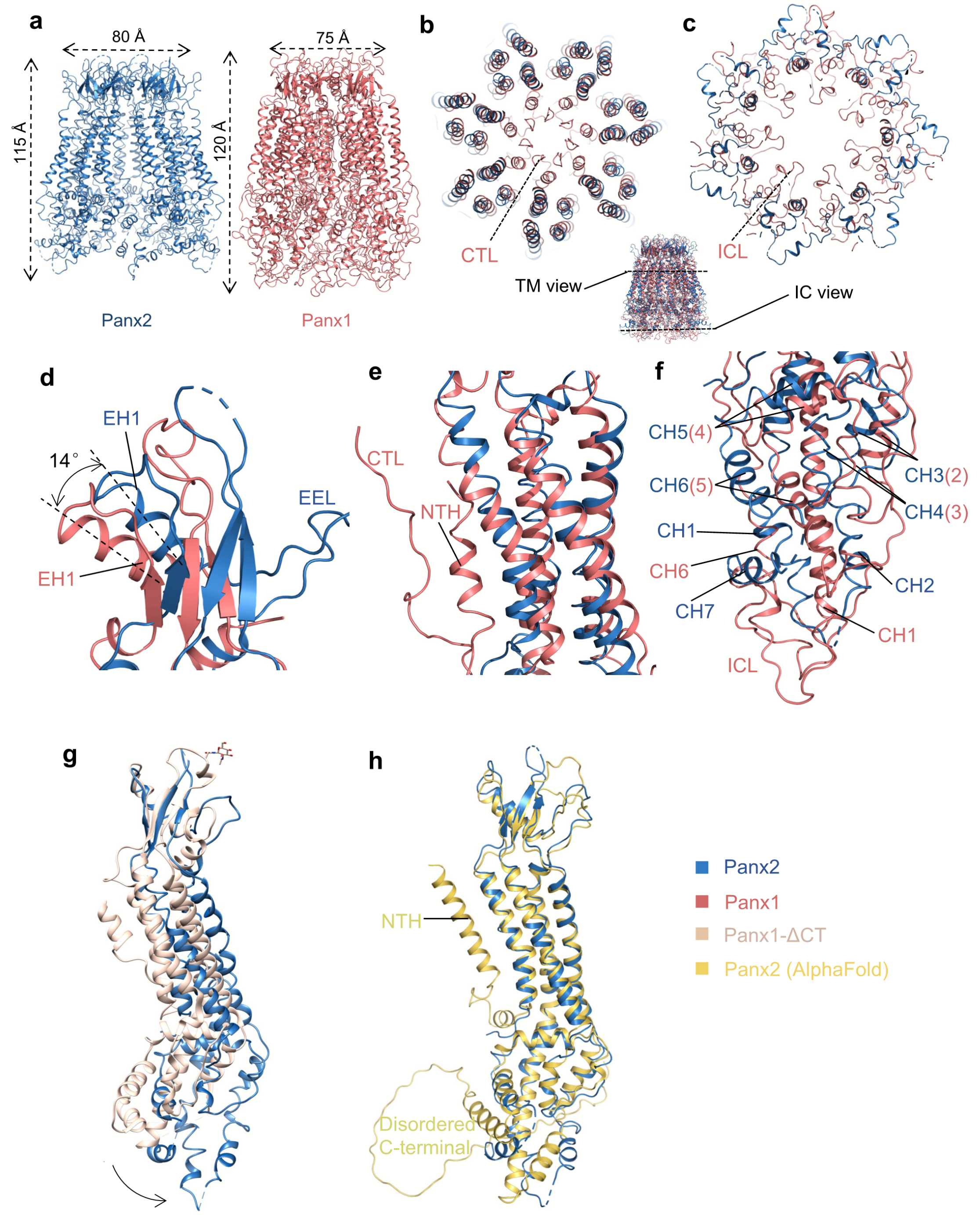

Fig. S5 Structure comparison of human Panx2 and Panx1.

All structures are displayed in ribbon representation. **a,** The Panx2 structure is colored in blue and the full-length Panx1 structure (PDB ID: 7DWB) is colored in deepsalmon. The width of extracellular side and length are indicated in double-arrowed dashed line. **b, c,** The transmembrane (TM) view **(b)** and the intracellular (IC) view **(c)** of the structural alignment of the Panx2 and full-length Panx1. The intracellular loop (ICL) and C-terminal loop (CTL) are indicated by dashed lines. **d, e, f,** The structural superposition between the Panx2 and Panx1 protomers viewed from the extracellular domain **(d)**, transmembrane domain **(e)** and intracellular domain **(f)**. EHs: extracellular helices, EEL: the extra extracellular loop, NTH: N-terminal helix, CHs: C-terminal helices. **g,** The structural superposition of protomers from the Panx2 and C-terminal truncated Panx1 (Panx1-ΔCT)

(PDB ID: 6WBG, wheat) with extracellular domain (ECD) fixed. **h**, The structural superposition of protomers from the Panx2 and AlphaFold predicted Panx2 (ID: AF-Q96RD6-F1, yellow). The partial C-terminal (amino acids 440-667) of AlphaFold predicted Panx2 is omitted for clarity. The NTH, ICL, CTL, CH1-CH6 of Panx1, EEL, CH1-CH7 of Panx2 and the NTH, disordered C-terminal of AlphaFold predicted Panx2 are highlighted.

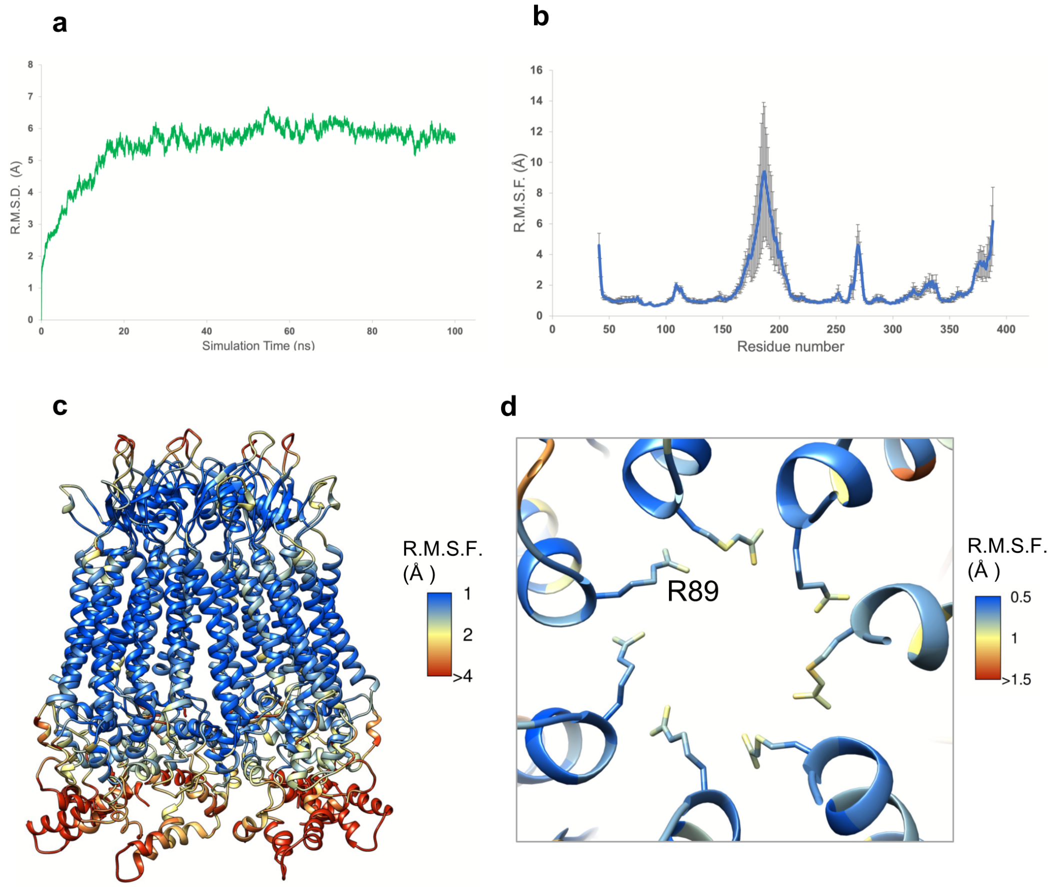

Fig S6 Analysis of protein backbone and R89 side chains dynamics during molecular dynamics simulations.

a, C_α root-mean-square deviation (R.M.S.D.) analysis of the molecular dynamics simulations, calculated with respect to the experimental starting structure for Panx2 model. **b,** Plot of average C_α root-mean-square fluctuation (R.M.S.F.) during the molecular dynamics simulations for Panx2. Averages are determined for the 7 subunits composing the channel. Error bars represent 95% confidence intervals (n = 7 subunits). **c,** Average R.M.S.F. values of the Panx2 model mapped to the experimental starting structure of Panx2. Colors correspond to R.M.S.F. amplitudes: 0-1.0 Å (bule); 1.0-2.0 Å (bule–yellow); 2.0-4.0 Å (yellow-red), >4.0 Å (red). **d,** Average R.M.S.F. values of the extracellular entrance of Panx2 model mapped to the experimental starting structure of Panx2. The side chains of R89 residues are presented. Colors correspond to R.M.S.F. amplitudes: 0-0.5 Å (bule); 0.5-1.0 Å (bule-yellow); 1.0-1.5 Å (yellow-red); >1.5 Å (red). Source data are provided as a Source Data file for panel **a** and **b**.

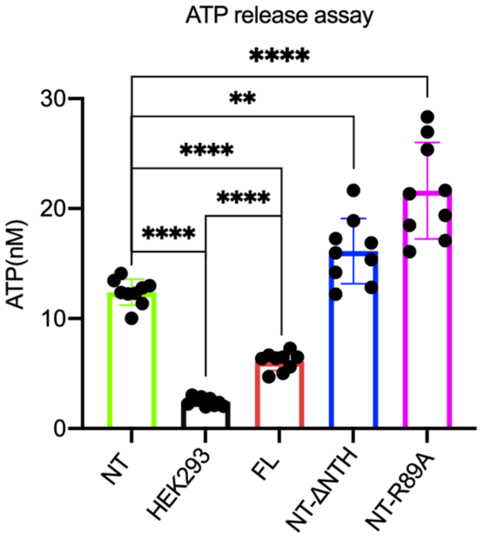

Fig. S7 Unnormalized ATP release assay.

The extracellular ATP concentrations were measured in adhered HEK293F cells transfected with full-length Panx2 (FL), Panx2-NT (NT), Panx2-NT-ΔNTH (NT-ΔNTH) or Panx2-NT-R89A (NT-R89A) plasmid. Control experiment (HEK293) was setup without any plasmid transfection. Bars indicate means ± SEM (n = 9 biologically independent dishes of cells). For statistical comparisons to NT or HEK293, the Student's t-test was applied (two-sided). P = 0.0029 (NT/NT-ΔNTH). ns, non-significant, *P<0.05, **P < 0.01, ***P < 0.001 and ****P < 0.0001. Source data are provided as a Source Data file.

Table S1 Cryo-EM data collection, refinement and validation statistics

	Panx2 (EMD-33276, PDB 7XLB)
**Data collection and processing**	
Magnification	130,000
Voltage (kV)	300
Electron exposure (e^{-}/Å^{2})	50
Defocus range (μm)	-1.0 to -2.5
Pixel size (Å)	0.92
Symmetry imposed	C7
Initial particle images (no.)	396,420
Final particle images (no.)	108,265
Map resolution (Å)	3.4
FSC threshold	0.143
Map resolution range (Å)	2.0 - 8.5
**Refinement**	
Initial model used (Alphafold entry)	Q96RD6
Model resolution (Å)	3.7
FSC threshold	0.5
Model resolution range (Å)	3.4 - 3.7
Map sharpening B factor (Å^{2})	-123.8
Model composition	
Nonhydrogen atoms	19,138
Protein residues	2532
Ligands	0
B factors (Å^{2})	
Protein	34.45
Ligand	N/A
R.m.s. deviations	
Bond lengths (Å)	0.003
Bond angles (°)	0.594
Validation	
MolProbity score	1.78
Clashscore	5.60
Poor rotamers (%)	0.67
Ramachandran plot	
Favored (%)	93.38
Allowed (%)	6.62
Disallowed (%)	0.00
