# Supplementary material for: Cryo-EM structure of human heptameric pannexin 2 channel
Source: Nat Commun. 2023 Mar 3;14:1118. doi: 10.1038/s41467-023-36861-x (PMC9984531; doi:10.1038/s41467-023-36861-x)
Supplement: Supplementary file 3 — Reporting Summary [file 41467_2023_36861_MOESM3_ESM.pdf]

nature portfolio

Corresponding author(s): Huawei Zhang

Last updated by author(s): Feb 1, 2023

Reporting Summary

Nature Portfolio wishes to improve the reproducibility of the work that we publish. This form provides structure for consistency and transparency in reporting. For further information on Nature Portfolio policies, see our Editorial Policies and the Editorial Policy Checklist.

Statistics

For all statistical analyses, confirm that the following items are present in the figure legend, table legend, main text, or Methods section.

n/a	Confirmed	
☐	☒	The exact sample size (*n*) for each experimental group/condition, given as a discrete number and unit of measurement
☐	☒	A statement on whether measurements were taken from distinct samples or whether the same sample was measured repeatedly
☐	☒	The statistical test(s) used AND whether they are one- or two-sided<br>*Only common tests should be described solely by name; describe more complex techniques in the Methods section.*
☒	☐	A description of all covariates tested
☒	☐	A description of any assumptions or corrections, such as tests of normality and adjustment for multiple comparisons
☐	☒	A full description of the statistical parameters including central tendency (e.g. means) or other basic estimates (e.g. regression coefficient) AND variation (e.g. standard deviation) or associated estimates of uncertainty (e.g. confidence intervals)
☐	☒	For null hypothesis testing, the test statistic (e.g. *F*, *t*, *r*) with confidence intervals, effect sizes, degrees of freedom and *P* value noted<br>*Give P values as exact values whenever suitable.*
☒	☐	For Bayesian analysis, information on the choice of priors and Markov chain Monte Carlo settings
☒	☐	For hierarchical and complex designs, identification of the appropriate level for tests and full reporting of outcomes
☒	☐	Estimates of effect sizes (e.g. Cohen's *d*, Pearson's *r*), indicating how they were calculated

Our web collection on statistics for biologists contains articles on many of the points above.

Software and code

Policy information about availability of computer code

Data collection	Cryo-EM: SerialEM(v3.5)
Data analysis	CryoEM Image Analysis Software: MotionCor2(v1.3.1), CTFFIND(v4.1), Relion(v3.1.2), Gautomatch(v0.56), Cryosparc(v3.3.1); Atomic modeling and visualization: Coot(v0.8.9), Phenix(v1.20.1), Molprobility(v4.5), Chimera(v1.16), PyMOL(v2.5.2), CAVER(v3.0.3); InterProSulf (http://curie.utmb.edu/prosurf.html, No version number issued by the developer); Microplate Reader: Gen5(v2.0); Data Analysis for Western Blot: ImageJ(v1.53k); Statistical Analysis: GraphPad Prism(v9.0.0); Molecular dynamics simulations: Prime and PROPKA modules from Schrodinger software (v2018), CHARMM-GUI (v3.8), PPM(v2.0), Gromacs (v2021),

For manuscripts utilizing custom algorithms or software that are central to the research but not yet described in published literature, software must be made available to editors and reviewers. We strongly encourage code deposition in a community repository (e.g. GitHub). See the Nature Portfolio guidelines for submitting code & software for further information.

Data

Policy information about availability of data

All manuscripts must include a data availability statement. This statement should provide the following information, where applicable:

- Accession codes, unique identifiers, or web links for publicly available datasets
- A description of any restrictions on data availability
- For clinical datasets or third party data, please ensure that the statement adheres to our policy

The cryo-EM map of human Panx2 was deposited in the Electron Microscopy Data Bank with accession code EMD-33276 [https://www.ebi.ac.uk/pdbe/entry/emdb/EMD-33276] (Cryo-EM structure of human pannexin 2). Atomic coordinates for the human Panx2 structure have been deposited in the Protein Data Bank with accession code 7XLB [https://doi.org/10.2210/pdb7LXB/pdb] (Cryo-EM structure of human pannexin 2). The source data underlying Figures 3b, 5c, 5d and Supplementary Figure 1b, 1d, 6a, 6b, 7 are provided as a Source Data file. Atomic coordinates for the human Panx1 structures could be accessed with PDB codes 6WBG [https://doi.org/10.2210/pdb6WBG/pdb] and 7DWB [https://doi.org/10.2210/pdb7DWB/pdb]. A computed model of human Panx2 was downloaded from the AlphaFold Protein Structure Database with ID: AF-Q96RD6-F1 [https://alphafold.ebi.ac.uk/entry/Q96RD6].Details for molecular dynamics simulations has been deposited in github at https://github.com/shiyu-wangbyte/panx2-simulation.

Human research participants

Policy information about studies involving human research participants and Sex and Gender in Research.

Reporting on sex and gender	N/A
Population characteristics	N/A
Recruitment	N/A
Ethics oversight	N/A

Note that full information on the approval of the study protocol must also be provided in the manuscript.

Field-specific reporting

Please select the one below that is the best fit for your research. If you are not sure, read the appropriate sections before making your selection.

☒ Life sciences ☐ Behavioural & social sciences ☐ Ecological, evolutionary & environmental sciences

For a reference copy of the document with all sections, see nature.com/documents/nr-reporting-summary-flat.pdf

Life sciences study design

All studies must disclose on these points even when the disclosure is negative.

Sample size	Cryo-EM sample size were not predetermined. An initial dataset of 1,476 micrographs (341,718 particles, Dataset 1) was collected, and two additional datasets of 5,030 micrographs (925,025 particles, Dataset 2) and 1,533 micrographs (345,848 particles, Dataset 3) were further collected and used to assess the effects of particle number on the achievable resolution and/or resolvability of particle features during 3D classification. The initial size of dataset 1 was estimated based on the particle density observed in test images. The increased size of dataset 3 vs. dataset 1 & 2 did not improve the global resolution of the resulting CryoEM maps, indicating the size of the particle datasets were sufficiently sampled. In general, total 8039 micrographs were collected for Cryo-EM and the sample size was sufficient as increased size did not further improve the final resolution and the resolution is good enough for further atomic model building.<br>For ATP release assay, each group was repeated for nine times. For Western Blot, each group was repeated for three times. Calculation of sample size for ATP release assay and Western Blot was based on similar experiments from previous reports and not predetermined.
Data exclusions	Single particle image data was excluded based on the absence of high-resolution features (e.g. alpha-helical transmembrane domains), which were conditions that had been pre-established based on expected structural homology to pannexin 1.<br>For ATP release assay, the minimum and maximum of every measurement were removed.<br>No data were excluded for Western Blot.
Replication	All attempts at replication were successful. This included processing three independent datasets obtained from unique particles in dataset 1 , dataset 2 and dataset 3, and by processing with alternative Cryo-EM image analysis software (Relion (v3.1.2)).<br>For ATP release assay, every group was repeated nine times.<br>For Western Blot, each group wad repeated three times.
Randomization	Single particle image data was split randomly into two groups and processed in the same way to calculate Fourier-shell correlation coefficients, in accordance to Gold Standard Methods. Samples were not further allocated into groups, outside of what is performed by the computational image analysis programs used in this work.

	For ATP release assay and Western Blot, cells ere randomly allocated into different experimental groups before transfected with different Panx2 constructs.
Blinding	Investigators were not blinded during data acquisition, experiment conduction or analysis. Blinded studies in this case were not possible because the investigator performed the experiments and analysis contributed by the isolation of the specimen or cells.

Reporting for specific materials, systems and methods

We require information from authors about some types of materials, experimental systems and methods used in many studies. Here, indicate whether each material, system or method listed is relevant to your study. If you are not sure if a list item applies to your research, read the appropriate section before selecting a response.

Materials & experimental systems

n/a	Involved in the study
☐	☒ Antibodies
☐	☒ Eukaryotic cell lines
☒	☐ Palaeontology and archaeology
☒	☐ Animals and other organisms
☒	☐ Clinical data
☒	☐ Dual use research of concern

Methods

n/a	Involved in the study
☒	☐ ChIP-seq
☒	☐ Flow cytometry
☒	☐ MRI-based neuroimaging

Antibodies

Antibodies used	Mouse anti-FLAG (Sino Biological Inc., Monoclonal Mouse IgG2a Clone #13, 109143-MM13, 1:1000).<br>Mouse anti-IgGκ secondary antibody conjugated with horseradish peroxidase (HRP) (Santa Cruz Biotechnology, sc-516102, 1:5000)
Validation	Mouse anti-FLAG (Sino Biological Inc., Monoclonal Mouse IgG2a Clone #13, 109143-MM13, 1:1000). Host species- mouse, reacts with: species independent; suitable for WB, ELISA, FCM, ICC/IF (https://cn.sinobiological.com/antibodies/flag-109143-mm13).

Eukaryotic cell lines

Policy information about cell lines and Sex and Gender in Research

Cell line source(s)	HEK293F cell (Thermo Fisher) and HEK293T cell (Thermo Fisher). HEK293F cells (Thermo Fisher, R79007)<br>HEK293T cells (Thermo Fisher, K1711)
Authentication	Cell lines were not authenticated.
Mycoplasma contamination	Cell lines were not tested for Mycoplasma contamination.
Commonly misidentified lines<br>(See ICLAC register)	No commonly misidentified lines were used in this study.
